# Effects of nitrogen stress on the photosynthetic CO_2_ assimilation, chlorophyll fluorescence, and sugar-nitrogen ratio in corn

**DOI:** 10.1038/srep09311

**Published:** 2015-04-01

**Authors:** Xiuliang Jin, Guijun Yang, Changwei Tan, Chunjiang Zhao

**Affiliations:** 1 Beijing Research Center for Information Technology in Agriculture, Beijing Academy of Agriculture and Forestry Sciences, Beijing, P.R. China; 2 Key Laboratory of Crop Genetics and Physiology of Jiangsu Province, Yangzhou University, Yangzhou 225009, P.R. China

## Abstract

A field experiment was conducted using three corn cultivars (Jingyu7, Nongda80, and Tangyu10) and three nitrogen (N) application rates (0, 75, and 150 kg N ha^−1^). The objectives of this study were to investigate the responses of photosynthetic CO_2_ assimilation (Ph), the maximum quantum yield of photosystem II (F_v_/F_m_), leaf dry weight (LDW), leaf nitrogen concentration (LNC), leaf sugar concentration (LSC), and the sugar-to-nitrogen concentration ratio (S/N) to N levels in three different field-grown corn cultivars on three sampling dates. The results showed that the LDW, F_v_/F_m_, Ph, LNC, and LSC increased with increasing N levels, and the variation patterns of F_v_/F_m_, Ph, and LNC were “low-high-low”. In contrast, S/N decreased with increasing N levels, and its variation pattern was “high-low-high”. The values of LDW, F_v_/F_m_, Ph, LNC, LSC, and S/N were greatest under high N conditions, followed by medium N conditions, and finally low N conditions. Significant interactions occurred between Ph, F_v_/F_m_, LNC, LSC, LDW, and S/N, with the exception of the interaction between LSC and S/N and between LSC and LDW. The correlation coefficients between Ph and S/N and between F_v_/F_m_ and S/N were −0.714 and −0.798, respectively.

Nitrogen (N) deficiency induces changes in many physiological processes^1^. For example, N deficiency significantly decreases the photosynthetic CO_2_ assimilation capacity of leaves, leading to decreases in light-saturated photosynthetic rates^2^,^3^,^4^,^5^ and photosynthetic quantum yields^6^. The decrease in the photosynthetic CO_2_ assimilation capacity is associated with decreases in the Rubisco content and activity of RuBPcase in the Calvin cycle^7^.

The photosynthetic capacity of leaves and their N content are positively correlated^3^,^8^,^9^. Increasing N concentrations in wheat plants can be an effective method to adjust the properties of photosynthetic pigments, improve the photosystem II (PSII) potential activity and maximum quantum yield, decrease non-photochemical quenching, and increase the net photosynthetic rate (Ph)^10^,^11^,^12^. Nitrogen deficiency decreases the quantum yield of PSII electron transport, CO_2_ assimilation of photosynthesis, and the maximal efficiency of PSII (F_v_/F_m_) photochemistry^13^,^14^,^15^. However, other researchers have shown that N deficiency has no effect on F_v_/F_m_, and thus results in no damage to the PSII^9^,^16^,^17^. For example, Nunes et al.^13^ showed that N deficiency had a large effect on the quantum yield of CO_2_ assimilation. However, Khamis et al.^9^ demonstrated that N deficiency had only a small effect on the quantum yield of CO_2_ assimilation, but it had a large effect on the light-saturated rate of photosynthesis. Nitrogen-deficient corn plants had a significantly smaller CO_2_ assimilation capacity, but did not differ from control plants with respect to the maximum efficiency of their PSII photochemistry (*F_v_*/*F_m_*)^18^. Zhang et al.^19^ suggested that the application of N fertilizer could significantly increase *F_v_*/*F_m_*. In their study, a high level of N fertilization increased the efficiency of the excitation energy captured by open PSII centers in certain wheat cultivar (SN1391) flag leaves. At the same time, the thermal dissipation decreased in wheat flag leaves receiving the high N treatment, but the decreased activity of the PSII resulted in the decline of the Ph in the flag leaves of certain wheat cultivars (GC8901). The results indicated that the effects of N application rates on the photosynthetic characteristics of flag leaves varied with wheat cultivars^20^.

In addition, studies have revealed that the total chlorophyll of leaves increases as the N supply increases^21^,^22^. Shrestha et al.^23^ showed that the F_v_/F_m_ differed significantly between two or more N levels. Most previous studies on the chlorophyll fluorescence and photosynthesis of corn leaves in response to N were conducted under artificial conditions. The results from studies using artificial conditions are not truly representative of field-grown corn environments. Therefore, it is necessary to measure the chlorophyll fluorescence and photosynthesis of corn leaves in response to N and analyze the relationships between chlorophyll fluorescence, photosynthesis, and leaf nitrogen concentration (LNC) in field-grown corn under different N levels. Although the sugar-to-nitrogen concentration ratio (S/N) better reflects the crop growth status than the LNC alone^24^, only a few field reports have investigated the relationships between chlorophyll fluorescence, photosynthesis, and S/N in field-grown corn. It is important to explain the relationships between chlorophyll fluorescence, photosynthesis, and S/N from the perspective of crop physiological processes, as this is useful for field corn management.

The objectives of this study were (i) to investigate Ph, F_v_/F_m_, LNC, leaf sugar concentration (LSC), leaf dry weight (LDW), and S/N in response to N application rates in different field-grown corn cultivars on three sampling dates, and (ii) to analyze the correlations between Ph, F_v_/F_m_, LNC, LSC, LDW, and S/N in leaves of different corn cultivars grown in the field. The results provide a practical basis for regulating N fertilizer application in corn and for breeding corn cultivars in China.

## Methods

### Experimental design

The field experiments were conducted from August to September during 2002 and 2003 at the Xiaotangshan experimental site (44.17**°**N, 116.433**°**E), Beijing, P.R. China. This area is representative of the overall soil and crop management in Beijing. The soil was fine-loamy with a nitrate-N (NO_3_-N) content of 16.7 ~ 18.03 mg kg^−1^, an ammonium-N (NH_3_-N) content of 10.2 ~ 12.3 mg kg^−1^, an Olsen P value of 15.2 ~ 17.6 mg kg^−1^, an exchangeable K content of 225 ~ 230 mg kg^−1^, and an organic matter content of 19.2 ~ 22.2 g kg^−1^ in the 0–30 cm soil layer.

Three local corn cultivars, Jingyu7 (JY7), Nongda80 (ND80), and Tangyu10 (TY10), were planted on June 25th, 2002 and June 27th, 2003. Nitrogen fertilizer (urea) was applied at three rates (0 (the low N treatment, LN), 75 (the medium N treatment, MN), and 150 (the high N treatment, HN) kg N ha^−1^) before planting, and the N application was distributed in three splits: 50% during the seeding stage, 25% during the vegetative 6 (V6) growth stage, and 25% during the vegetative tassel stage. For all treatments, 105 kg ha^−1^ of P_2_O_5_ (as monocalcium phosphate [Ca(H_2_PO_4_)_2_]) and 112.5 kg ha^−1^ of K_2_O (as KCl) were applied prior to the seeding. The size of each subplot was 15 m × 7 m. There were 2 m-wide buffer corridors between each subplot. The experiment was designed as a 2-way factorial arrangement of treatments in a randomized complete block design with three replications for each treatment. The other management elements followed the local standard practices of wheat production.

### Plant measurements

The aboveground biomass was destructively sampled by randomly cutting four representative plants from each plot. All the plant samples were heated to 105°C, oven dried at 70°C until a constant weight was achieved, and the samples were then weighed. The dry plant material was then ground so that it passed through a 240-mesh screen, and the ground samples were analyzed for total N using a Carlo Erba NA 1500 dry combustion analyzer (Carlo Erba, Milan, Italy)^36^. The quantitative analysis of the total soluble sugar content was measured with the anthrone colorimetric method^37^.

### Photosynthetic CO_2_ assimilation measurements

The Ph was measured with a portable leaf chamber and an open-system infrared gas analyzer (IRGA) (LI-6400; Li-Cor Inc., Lincoln, NE, USA). To minimize sources of diurnal heterogeneity, the measurements were only conducted during mid- and late- morning (usually 09:00–11:30) on uniformly sunlit days. The Ph was measured under ambient CO_2_ concentrations (approximately 370 μmol m^−2^ s^−1^) and a photosynthetic photon flux density (PPFD) of 1500 μmol m^−2^ s^−1^, provided by a red–blue light source (6400-02B). The ambient temperature ranged from 24 to 28°C and the temperature in the leaf chamber was about 25°C. The main function of the leaves (corn-cob leaves) was measured using the IRGA, with at least three replications. The Ph measurements were repeated five times in each subplot, for a total of five replications, which were averaged to represent the Ph of each subplot.

### Chlorophyll fluorescence measurements

Chlorophyll fluorescence was determined on leaf discs using a pulse amplitude modulation portable fluorometer (PAM-2100, Walz, Effeltrich, Germany). The initial fluorescence (F_o_) and maximum fluorescence (F_m_) were analyzed, and the F_v_/F_m_ was calculated. The leaf discs were adapted to the dark for 30 min prior to the measurements, so that all the PSII centers were open (i.e., all the primary acceptors were oxidized) and heat dissipation was minimum. The F_o_ was obtained with a low-intensity modulated light (<0.1 μmol m^−2^ s^−1^), so as not to induce any effect in the fluorescence variable. The F_m_ was obtained with 0.3-s pulses of saturated white light with an intensity of 14,000 μmol m^−2^ s^−1^. The fluorescence variable (F_v_) was calculated as the difference between F_m_ and F_o_. The F_v_ and F_m_ values were used to obtain the F_v_/F_m_. The F_v_/F_m_ was measured using the PAM-2100 while holding Ph at the same position, with at least three replications. The F_v_/F_m_ measurements were also repeated for five replications in each subplot and were averaged to represent the F_v_/F_m_ of each subplot.

### Statistical analysis

The standard errors, variance, regression and correlation coefficients, and significant differences among the regression coefficients were calculated using standard methods with SPSS software (16.0, SPSS, Chicago, IBM, USA). When it was necessary, the data was classified before the statistical analyses.

## Results

### Leaf dry weight (LDW)

**Figure 1:**
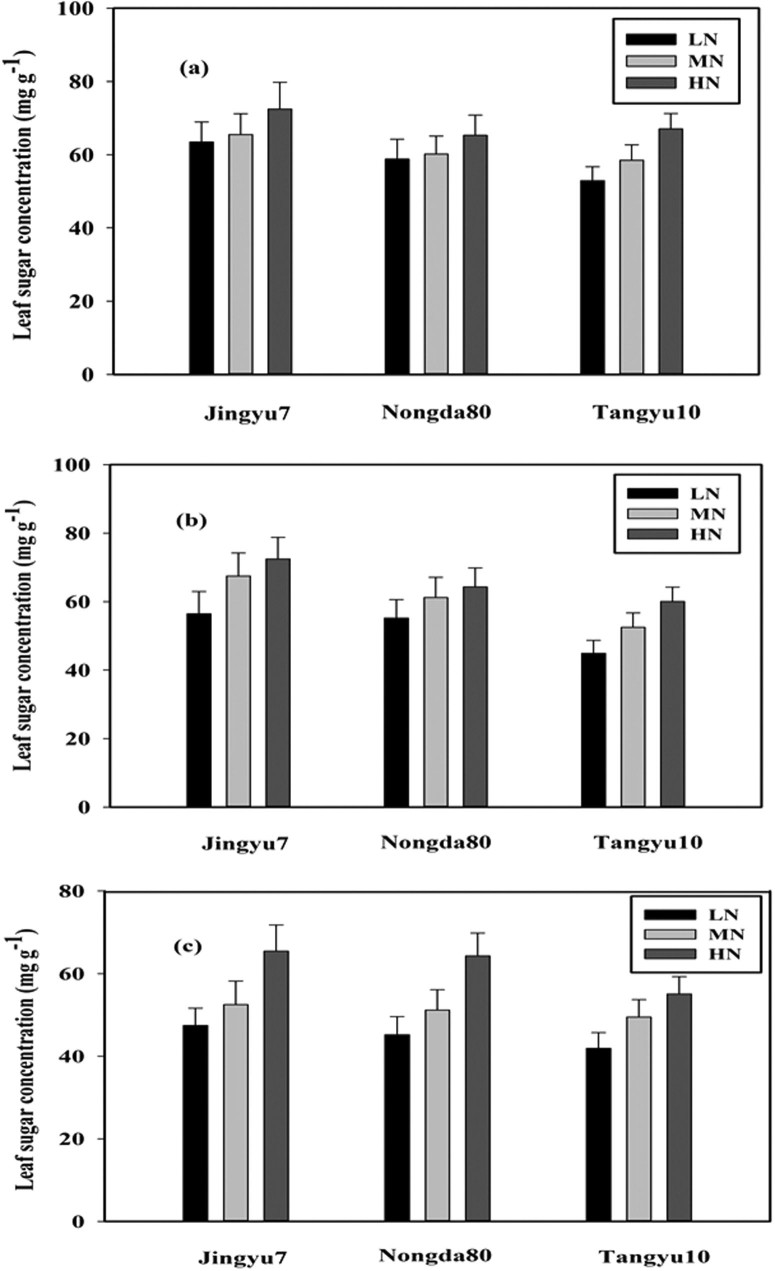
Leaf dry weight for August 16th (a), September 6th (b), and September 17th (c) of corn subjected to low (LN, 0 kg N ha^−1^), medium (MN, 75 kg N ha^−1^), and high (HN, 150 kg N ha^−1^) levels of N over the growing season (2002 and 2003). Data are the average values across 2 years with ± one standard error (vertical bars). Analysis of variance (ANOVA) results for (a) August 16th: nitrogen (N), P < 0.004; corn cultivars (C), P < 0.045; N × C, P = 0.271; (b) September 6th: N, P < 0.002; C, P < 0.009; N × C, P = 0.145; (c) September 17th: N, P < 0.036; C, P < 0.043; N × C, P = 0.345.

The aboveground LDW accumulation was analyzed for the three sampling dates with respect to the different N levels and corn cultivars (Figs. 1a, 1b, 1c and Table 1). We selected g m^−2^ (the mass of the leaves per square meter of soil) as the unit of LDW, as this unit better describes the crop growth status^38^. A significant difference between the different corn cultivars and N levels existed, with exception of the Nongda80 (ND80) cultivar. The results showed that the different N levels had a relatively small effect on the LDW accumulation in ND80. This indicated that the LDW accumulation in ND80 was not very sensitive to different N levels, which may be related to the wheat cultivar type. On August 16th, the LDW accumulation was greater under the HN treatment than under MN and LN treatments by 53.1% and 17.7% for the Jingyu7 (JY7) cultivar and by 60.1% and 29.0% for the Tangyu10 (TY10) cultivar, respectively. The LDW accumulation for the MN treatment was greater than that for the LN treatment by 30.1% and 24.0% for JY7 and TY10 on August 16th, respectively (Fig. 1a). Similarly, on September 6th and September 17th, the LDW accumulation was greatest under the HN treatment, followed by the MN treatment, and finally the LN treatment (Figs. 1b and 1c). With the development of growth stages, the LDW accumulation changed sharply with increasing N levels for JY7 and TY10, but little change in the LDW accumulation of ND80 was observed. The effects of the N applications and cultivars were significant for the three sampling dates; however, the interactions between the N applications and cultivars were not significant (Table 1).

### The maximum quantum yield of PSII (F_v_/F_m_)

The different N levels had a significant effect on the seasonal dynamics of the F_v_/F_m_ (Table 2). The JY7 and ND80 cultivars were significantly more sensitive to the different N levels than TY10. However, the effect of the N levels on TY10 was not significantly different between the HN and MN treatments, but it was significantly different between the MN and LN treatments on all three sampling dates (Tables 1 and 2). On August 16th, the F_v_/F_m_ was higher under the HN treatment than under the MN and LN treatments by 6.8% and 4.3%, 6.7% and 3.3%, and 6.2% and 2.6% for JY7, ND80, and TY10, respectively. The F_v_/F_m_ under the MN treatment was higher than that under the LN treatment by 2.4%, 3.3%, and 3.4% for JY7, ND80, and TY10 on August 16th, respectively. Similarly, the F_v_/F_m_ was higher under the HN treatment than under the MN and LN treatments, and the F_v_/F_m_ was higher under the MN treatment than under the LN treatment on September 6th and September 17th (Tables 1 and 2). The F_v_/F_m_ values of the different corn cultivars were significantly different on all three sampling dates, and the interactions effects between the N levels and cultivars were also significant, except on September 17th (Tables 1 and 2). The results suggested that the interaction effects between the N levels and cultivars were not significant because the corn leaves entered senescence on September 17th.

### Photosynthetic CO_2_ assimilation (Ph)

The Ph significantly increased with increasing N levels (Tables 1 and 3). On August 16th, the average Ph values under the HN treatment vs. the MN and LN treatments were 20.88 μmol m^−2^ s^−1^ vs. 26.69 μmol m^−2^ s^−1^ (+27.8%) and 33.7 μmol m^−2^ s^−1^ (+61.4%) for JY7; 18.26 μmol m^−2^ s^−1^ vs. 25.56 μmol m^−2^ s^−1^ (+40.0%) and 31.67 μmol m^−2^ s^−1^ (+73.4%) for ND80; and 17.94 μmol m^−2^ s^−1^ vs. 24.59 μmol m^−2^ s^−1^ (+37.1%) and 29.59 μmol m^−2^ s^−1^ (+64.5%) for TY10, respectively. The variations in Ph were similar to those in F_v_/F_m_ on September 6th and September 17th (Tables 1 and 3). The N levels exhibited a strong effect on the Ph values. Similarly, the interactions between the N levels and cultivars were significant, except on September 17th (Tables 1 and 3). This was because the corn leaves entered senescence, which caused the interactions between the N levels and cultivars to be non-significant.

### Leaf N concentration (mg g^−1^)

**Figure 2:**
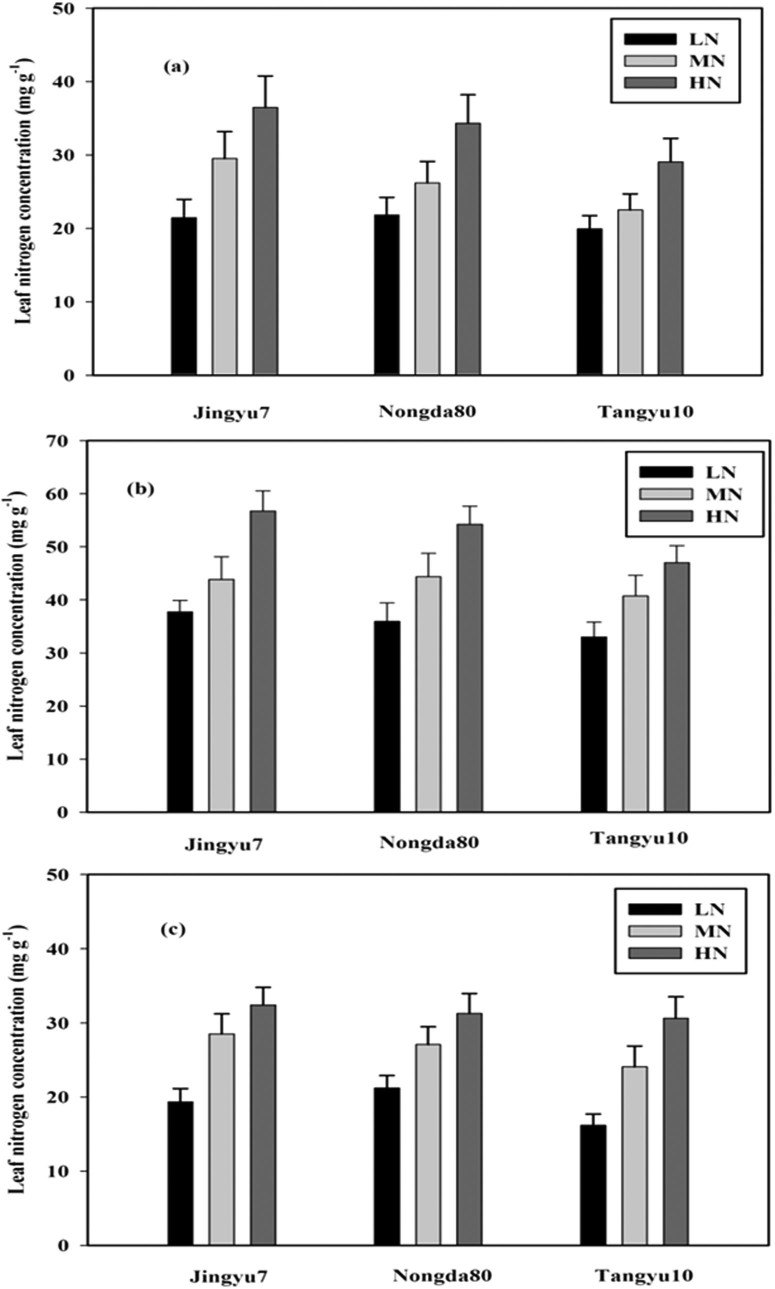
Leaf N concentration on August 16th (a), September 6th (b), and September 17th (c) of corn subjected to low (LN, 0 kg N ha^−1^), medium (MN, 75 kg N ha^−1^), and high (HN, 150 kg N ha^−1^) levels of N application over three cropping seasons (2002 and 2003). Data are average values across 2 years with ± one standard error (vertical bars). ANOVA results for (a) August 16th: nitrogen (N), P < 0.034; corn cultivars (C), P = 0.041; N × C, P = 0.865; (b) September 6th: N, P < 0.006; C, P = 0.035; N × C, P = 0.667; (c) September 17th: N, P < 0.041; C, P = 0.456; N × C, P = 0.766.

On August 16th, the mean LNC were 21.45 mg g^−1^, 29.5 mg g^−1^, and 36.47 mg g^−1^ for JY7; 21.82 mg g^−1^, 26.20 mg g^−1^, and 34.30 mg g^−1^ for ND80; and 19.92 mg g^−1^, 22.50 mg g^−1^, and 29.05 mg g^−1^ for TY10 under the LN, MN, and HN treatments, respectively (Fig. 2a). On September 6th, the mean LNC increased to its maximum values, which were 37.70 mg g^−1^, 43.84 mg g^−1^, and 56.75 mg g^−1^ for JY7; 35.93 mg g^−1^, 44.36 mg g^−1^, and 54.26 mg g^−1^ for ND80; and 33.05 mg g^−1^, 40.73 mg g^−1^, and 47.12 mg g^−1^ for TY10 under the LN, MN and HN treatments, respectively (Fig. 2b). However, on September 17th, the mean LNC dropped sharply to 19.33 mg g^−1^, 28.51 mg g^−1^, and 32.40 mg g^−1^ for JY7; 21.20 mg g^−1^, 27.08 mg g^−1^, and 31.24 mg g^−1^ for ND80; and 16.18 mg g^−1^, 24.08 mg g^−1^, and 30.61 mg g^−1^ for TY10 under the LN, MN, and HN treatments, respectively (Fig. 2c). The N levels significantly increased the LNC in the different corn cultivars throughout the crops' development, but the difference in the magnitude of the increase between the growth stages and corn cultivars was similar across N levels. On August 16th, the LNC was greater under the HN treatment than under the LN and MN treatments by 70.0% and 57.2%, 45.8% and 23.6%, and 30.9% and 29.1%, for JY7, ND80, and TY10, respectively. In addition, the LNC was greater under the MN treatment than under the LN treatment by 37.5%, 20.8%, and 12.9% for JY7, ND80, and TY10 (Fig. 2a). Similarly, on September 6th and September 17th, the LNC was greatest under the HN treatment, followed by the MN treatment, and finally by the LN treatment (Figs. 2b, 2c and Table 1). The effects of the N levels and cultivars on the LNC were significant across the growth stages, but the interaction effects between the N levels and cultivars were not significant (Table 1).

### Leaf sugar concentration (mg g^−1^)

**Figure 3:**
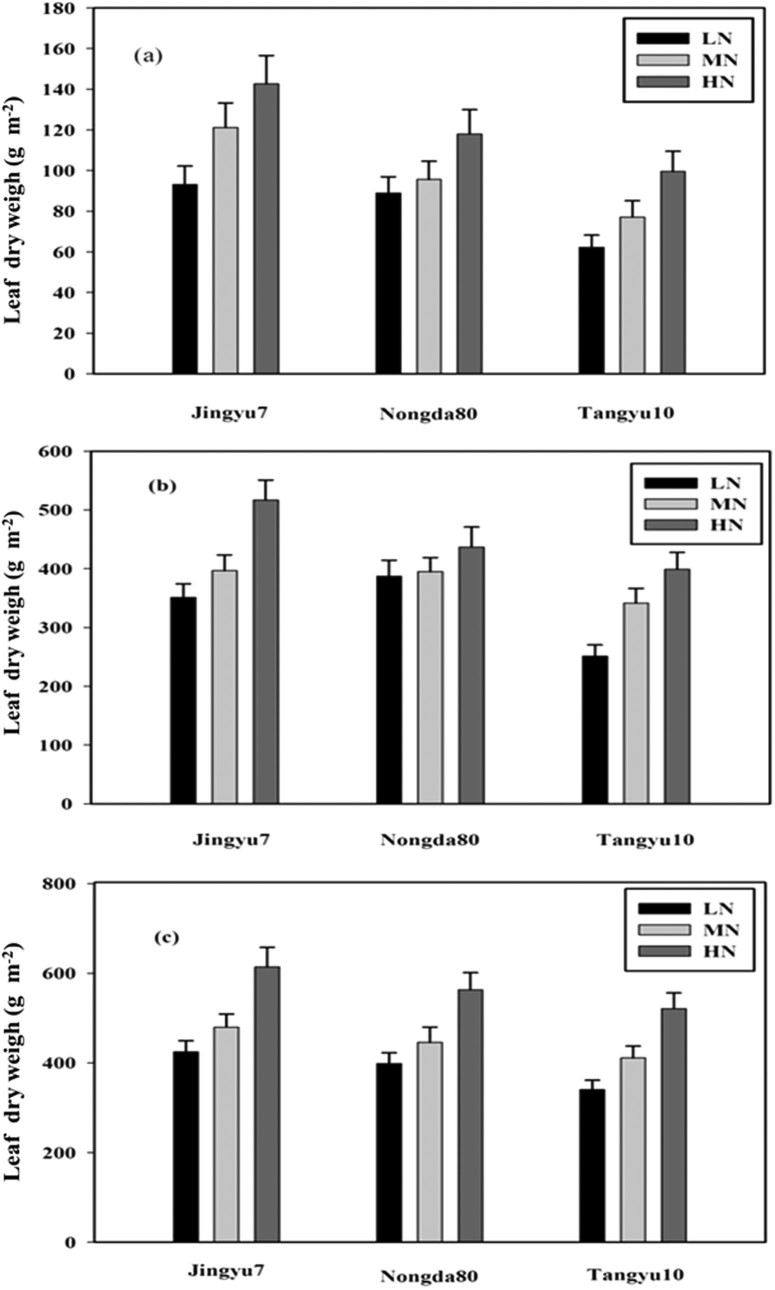
Leaf sugar concentration for August 16th (a), September 6th (b), and September 17th (c) of corn subjected to low (LN, 0 kg N ha^−1^), medium (MN, 75 kg N ha^−1^), and high (HN, 150 kg N ha^−1^) levels of N application over the cropping season (2002 and 2003). Data are average values across 2 years with ± one standard error (vertical bars). ANOVA results for (a) August 16th: nitrogen (N), P = 0.242; corn cultivars (C), P < 0.321; N × C, P = 0.664; (b) September 6th: N, P = 0.036; C, P < 0.431; N × C, P < 0.042; (c) September 17th: N, P = 0.032; C, P = 0.446; N × C, P = 0.862.

The mean LSC on September 17th was much greater than that on September 6th and August 16th. On August 16th, the mean LSC was greater than that on September 6th under the MN treatment, but less than that under the HN treatment. The LSC changed in response to N levels to a much less degree than LNC: averaged across N levels and corn cultivars, LSC increased under the MN and HN treatments by 10.8% and 18.4%, 6.83% and 20.9%, and 17.2% and 33.33% on August 16th, September 6^th^, and September 17th, respectively. The cultivar effects were not significant on the three sampling dates (Figs. 3a, 3b, 3c and Table 1). The N effects were significant for the LSC in all growth periods, but the interactions between the corn cultivars and N levels were not significant (Table 1).

### The sugar-to-nitrogen concentration ratio (S/N)

Exposure to N strongly decreased the S/N (Table 4), and N fertilization had a significantly positive effect on S/N (Tables 1 and 4). The changes in S/N were opposite to the changes in Ph and F_v_/F_m_; the S/N gradually decreased with increasing N levels and the development of growth stages (Table 4). The effects of the corn cultivars and N levels were not significant for S/N, and the interactions between the N levels and corn cultivars were also not significant.

### Interaction relationships between Ph, F_v_/F_m_, LNC, LSC, LDW, and S/N

There were highly significant (P < 0.01) interactions between Ph, F_v_/F_m_, LNC, LSC, LDW, and S/N, with the exception of the interaction between LSC and S/N and between LSC and LDW (Table 5). The results suggested that the LSC was relatively less sensitive to different N levels than LDW and S/N. Therefore, the interactions between LSC and S/N and between LSC and LDW were significant (P < 0.05).

The relationship between LDW and F_v_/F_m_ had the highest correlation coefficient (r = 0.909), followed by that between LNC and S/N (r = −0.886). The lowest r was between LSC and LNC (r = −0.408), which were significantly (P < 0.05) correlated. The remaining results from the correlation analyses are shown in Table 5. These results indicated that Ph and F_v_/F_m_ could be used to estimate LDW, LNC, LSC, and S/N; therefore, they can serve as good indicators for monitoring LDW, LNC, LSC, and S/N changes.

The LDW, F_v_/F_m_, Ph, LNC, and LSC increased in response to increased N levels and the variation patterns of F_v_/F_m_, Ph, and LNC were “low-high-low”. In contrast, S/N decreased as the N levels increased, and its variation pattern was “high-low-high”. The results showed that the different corn cultivars and N levels significantly influenced Ph, F_v_/F_m_, and S/N on the three sampling dates. The effects of the interactions between the N levels and cultivars on Ph, F_v_/F_m_, LDW, LNC, and LSC were significantly different, with the exception of Ph and F_v_/F_m_ on September 17th, but the interactions were not significantly different for S/N on the three sampling dates. Similarly, the interactions between the N levels and cultivars did not significantly affect Ph and F_v_/F_m_ based on the analysis of the Ph and F_v_/F_m_ values on September 17th. These results indicated that strong correlations existed between Ph and S/N and between F_v_/F_m_ and S/N, which were −0.714 and −0.798, respectively; the relationships between LNC and F_v_/F_m_ and between LNC and Ph were 0.636 and 0.671, respectively. This suggested that Ph and F_v_/F_m_ could be used to estimate S/N.

## Discussion

### Leaf dry weight

Leaf dry weight is formed by the conversion of solar energy into biomass, a process that partially depends on the N supply^25^,^26^,^27^. Accordingly, in our study, LDW increased with the increased N levels and the development of growth stages. The results were in agreement with those of Biemond et al.^28^,^29^ and Vos et al.^30^ However, for the same N levels, the different corn cultivars also had a significant effect on LDW, probably because a large difference in N utilization and distribution existed among the cultivars. The positive relationship between leaf chlorophyll content (LCC) and LNC^21^,^22^ indicates that the solar energy absorbed by chlorophyll was converted into biomass. Because this process is related to N supply and a large difference in N utilization and distribution existed among the corn cultivars', the different LNC corresponded to the different LCC, and the different LCC led to the different LDW in the different corn cultivars at the same N levels.

### Ph and F_v_/F_m_

In this study, the Ph and F_v_/F_m_ increased as the N levels increased (Tables 2 and 3), and these results were consistent with those of Kao et al.^31^ and Shang guan et al.^32^ However, the N applications significantly increased the F_v_/F_m_ on the three sampling dates (Table 3), which was not in agreement with the results of Lu et al.^18^, but was consistent with those Zhang et al.^19^ and Shrestha et al.^23^. The former two studies suggested that the corn cultivars contained abundant chlorophyll under HN levels, and thus the PSII apparatus remained functional, the crops had lots of green leaves, and there was a continued use of the light captured by the remaining PSII apparatus, leading to a higher Ph, F_v_/F_m_, LCC, and PSII efficiency. However, under LN levels, many of the green leaves became yellow, and the PS II apparatus captured less light, causing lower Ph and F_v_/F_m_ values. Under different N levels, the corn cultivars had a significant effect on the Ph and F_v_/F_m_, which was due to the significant effect of the corn cultivars on the LNC (Figs. 2a, 2b, 2c, Tables 2 and 3). The relatively higher LNC corresponded to the relatively higher Ph and F_v_/F_m_ because, as some studies have shown, LCC and LNC are highly positively correlated^21^,^22^. The main reasons for this were the following: (1) LCC had a close relationship with photosynthetic electron transport, which directly affected the Ph and F_v_/F_m_^18^,^19^, with higher LCC resulting in higher Ph and F_v_/F_m_; (2) LNC strongly affected LCC^23^, LNC had good relationships with Ph and F_v_/F_m_ (Table 5), and LCC and LNC were influenced by different N levels^20^,^23^; and (3) higher LCC and LNC values were linked to higher Ph and F_v_/F_m_ values because these parameters increased the Rubisco content and activity of RuBPcase in corn plants, thereby improving corn chlorophyll fluorescence and photosynthesis. Therefore, different the N levels influenced the changes in Ph and F_v_/F_m_ (Table 1, 2 and 3).

### Leaf sugar concentration

Leaf sugar concentration (LSC) is an important indicator of physiological changes in crops. The LSC increased with increasing N levels (Figs. 3a, 3b, and 3c). Hence, LNC could be used to improve the LSC in corn cultivars, which is in agreement with the findings of Sun et al.^33^ and Feng et al.^24^ However, the LSC among the different corn cultivars did not differ because the corn cultivars were less sensitive to LSC than LNC (Table 1). Over the development of growth stages, the LSC and LNC first increased gradually until the vegetation tasseling stage; then, the LSC and LNC decreased gradually until maturity, owing to the allocation of the LSC and LNC to the corn grains.

### Sugar-nitrogen concentration ratio (S/N)

The effect of N on the S/N was the opposite to that of the LNC and LSC. The S/N significantly decreased with increasing N levels. The S/N significantly changed with the development of growth stages, which was consistent with the results of Tian et al.^34^, and the variation pattern of S/N was “high-low-high” (Table 4). The main reasons for this were as follows. (1) A positive relationship existed between LNC and LSC (Table 5). (2) Differences in the speed of LNC and LSC production existed owing to the different growth stages of corn. LSC was higher than LNC on August 16th because the corn plants were relatively small and needed more LSC to protect their normal growth from extreme environmental changes; however, over the development of its growth stages, the corn needed to increase the LNC more than the LSC for vegetation growth because the crops needed more photosynthesis to maintain their normal growth levels. Thus, the speed of LNC production was faster that of LSC, and consequently, LNC was higher than LSC on September 6th. During its later growth stages, the corn required more LNC than LSC to form corn grains and maintain its photosynthetic functions, and thus LSC was higher than LNC on September 17th. (3) The LNC and LSC changes were used to satisfy the needs of crop growth^24^ and maintain the balance of photochemistry and other physiological functions^33^,^34^. Therefore, S/N exhibited a “high-low-high” variation pattern (Table 4). The S/N was also significantly different among the different N levels (Table 1), suggesting that the different N levels also influenced the S/N levels.

### Correlations between Ph, F_v_/F_m_, LNC, LSC, LDW, and S/N

The interactions between Ph, F_v_/F_m_, LNC, LSC, LDW, and S/N were highly significant (P < 0.01), except the interactions between LSC and S/N and between LSC and LDW. A significant relationship existed between LDW and F_v_/F_m_ (r = 0.909), which may have been related to the strong relationship between N and F_v_/F_m_^13^,^14^. The main reasons for this were (1) the LCC was strongly affected by the N concentration, and thus the F_v_/F_m_ were influenced by the increased N levels^19^, leading to an increase in chlorophyll concentration and LDW; (2) the chlorophyll concentration and LDW also had a highly significant relationship^30^; (3) the increase in F_v_/F_m_ is associated with the increase in Rubisco content, and the activity of RuBPcase in the Calvin cycle and phosphoenolpyruvate carboxylase (also known as PEP carboxylase) in the C_4_ photosynthetic path can be used to improve the photosynthetic electron transport in chloroplasts, yielding oxygen, nicotinamide adenine dinucleotide phosphate hydrate (NADPH), and ATP, thereby increasing photosynthesis, chlorophyll concentration, and thus elevating the LDW of corn plants. Therefore, the correlation coefficient between LDW and F_v_/F_m_ was high. Similarly, a close relationship between Ph and LDW existed: the higher N concentrations increased the RuBPcase and PEP carboxylase activity, thereby improving the carbon dioxide fixation ability and increasing the Ph of corn plants; thus, the LDW increased with the increased Ph. The results also demonstrated that the LSC influenced the changes of Ph and F_v_/F_m_ (Table 5). Strong relationships existed between the sugar concentration and Ph, F_v_/F_m_, LNC, LSC, and LDW; this was because sugar performs a supporting role in increasing Ph, F_v_/F_m_, LNC, LSC, and LDW. Negative correlations existed between S/N and LDW, F_v_/F_m,_ Ph, N, and S, which may be due to the fact that S/N exhibited an opposite variation pattern to LDW, F_v_/F_m,_ Ph, LNC, and LSC.

As the correlation coefficients between Ph and S/N and between F_v_/F_m_ and S/N were −0.714 and −0.798, respectively, Ph and F_v_/F_m_ could be used to estimate S/N (Tables 1 and 5). The correlation coefficients between LNC and F_v_/F_m_ and between LNC and Ph were 0.636 and 0.671, respectively, which were in agreement with the results of Wu^35^. These results suggested that the relationships between Ph, F_v_/F_m_, and S/N were better than those between Ph, F_v_/F_m_, and LNC; therefore, the Ph and F_v_/F_m_ were better estimates of S/N.

## Conclusion

The results of this study showed that the LDW, F_v_/F_m_, Ph, LNC, and LSC increased with increasing N levels in different field-grown corn cultivars on three sampling dates, and the variation patterns of F_v_/F_m_, Ph, and LNC were “low-high-low”. In contrast, S/N decreased with increasing N levels, and its variation pattern was “high-low-high”. The values of LDW, F_v_/F_m_, Ph, LNC, LSC, and S/N were the greatest under high nitrogen (HN) conditions, followed by medium nitrogen (MN) conditions, and finally low nitrogen (LN) conditions. Significant (P < 0.01) interactions occurred between Ph, F_v_/F_m_, LNC, LSC, LDW, and S/N, with the exception of the interaction between LSC and S/N and between LSC and LDW. The good correlation coefficients existed between Ph and S/N and between F_v_/F_m_ and S/N, which were −0.714 and −0.798, respectively; the correlation coefficients between LNC and F_v_/F_m_ and between LNC and Ph were 0.636 and 0.671, respectively.

## Figures and Tables

**Table 1:** Effects of N and corn cultivars on corn growth

Parameters	Stages or periods	N	C	C × N
**Leaf dry weight (g m^−2^)**	A	**	*	n.s.
	B	**	**	n.s.
	C	*	*	n.s.
**The maximum quantum yield of PSII (F_v_/F_m_)**	A	*	*	*
	B	*	*	*
	C	*	*	n.s.
**Photosynthetic CO_2_ assimilation (Ph, μmol m^−2^ s^−1^)**	A	**	*	*
	B	**	**	**
	C	**	*	n.s.
**Leaf N concentration (mg g^−1^)**	A	*	*	n.s.
	B	**	*	n.s.
	C	*	n.s.	n.s.
**Leaf sugar concentration (mg g^−1^)**	A	n.s.	n.s.	n.s.
	B	*	n.s.	n.s.
	C	*	n.s.	n.s.
**The sugar-to-nitrogen concentration ratio (%)**	A	*	n.s.	n.s.
	B	*	n.s.	n.s.
	C	*	n.s.	n.s.

Note:^a^A, B, and C denote three successive growth stages: August 16th, September 6th, and September 17th, respectively.

bProbability levels are indicated by n.s., *, and ** for ‘not significant’, P < 0.05, and P < 0.01, respectively.

**Table 2:** Effect of N on the maximum quantum yield of PSII (F_v_/F_m_) in different corn cultivars

Corn cultivars	Treatment	August 16th	September 6th	September 17th
**Jingyu7**	LN	0.709 ± 0.022 cC	0.815 ± 0.007 cC	0.796 ± 0.005 cB
	MN	0.726 ± 0.015 bB	0.826 ± 0.008 bB	0.812 ± 0.006 bA
	HN	0.757 ± 0.005 aA	0.835 ± 0.006 aA	0.824 ± 0.007 aA
**Nongda80**	LN	0.702 ± 0.013 cC	0.812 ± 0.011 cC	0.793 ± 0.009 bB
	MN	0.725 ± 0.012 bB	0.824 ± 0.009 bB	0.815 ± 0.006 aA
	HN	0.749 ± 0.015 aA	0.837 ± 0.011 aA	0.822 ± 0.004 aA
**Tangyu10**	LN	0.695 ± 0.017 bB	0.782 ± 0.022 bB	0.755 ± 0.011 bB
	MN	0.719 ± 0.012 aA	0.816 ± 0.012 aA	0.792 ± 0.019 aA
	HN	0.738 ± 0.007 aA	0.825 ± 0.007 aA	0.817 ± 0.009 aA

Note: The statistical analyses are based on each cultivar. With respect to each cultivar, the values in the same column followed by different uppercase and lowercase letters are significantly different at the 0.01 and 0.05 probability levels, respectively. The corn cultivars were subjected to low (LN, 0 kg N ha^−1^), medium (MN, 75 kg N ha^−1^), and high (HN, 150 kg N ha^−1^) levels of N application over the cropping season (2002 and 2003). The data are average values across 2 years with ± one standard error.

**Table 3:** Effect of N on photosynthetic CO_2_ assimilation (Ph, μmol m^−2^ s^−1^) in different corn cultivars

Corn cultivars	Treatment	August 16th	September 6th	September 17th
**Jingyu7**	LN	20.88 ± 0.57cC	22.94 ± 0.64 cC	16.64 ± 0.54 cC
	MN	26.69 ± 0.45 bB	34.69 ± 0.98 bB	24.69 ± 0.62 bB
	HN	33.70 ± 0.88 aA	42.32 ± 1.06 aA	36.72 ± 1.43 aA
**Nongda80**	LN	18.26 ± 0.66 cC	26.26 ± 0.011 cC	19.24 ± 0.39 cC
	MN	25.56 ± 0.85 bB	33.43 ± 0.74 bB	27.12 ± 0.48 bB
	HN	31.67 ± 0.75 aA	41.43 ± 0.42 aA	34.45 ± 0.68 aA
**Tangyu10**	LN	17.94 ± 0.67 cC	21.64 ± 0.42 cC	14.46 ± 0.24 cC
	MN	24.59 ± 0.42 bB	32.55 ± 0.87 bB	22.76 ± 0.74 bB
	HN	29.59 ± 0.67 aA	40.21 ± 1.13 aA	32.43 ± 0.68 aA

Note: The statistical analyses are based on each cultivar. With respect to each cultivar, the values in the same column followed by different uppercase and lowercase letters are significantly different at the 0.01 and 0.05 probability levels, respectively. The corn cultivars were subjected to low (LN, 0 kg N ha^−1^), medium (MN, 75 kg N ha^−1^), and high (HN, 150 kg N ha^−1^) levels of N application over the cropping season (2002 and 2003). The data are average values across 2 years with ± one standard error.

**Table 4:** Effect of N on the sugar-to-nitrogen concentration ratio (S/N) in different corn cultivars

Corn cultivars	Treatment	August 16th	September 6th	September 17th
**Jingyu7**	LN	2.96 ± 0.27aA	1.57 ± 0.14 aA	2.51 ± 0.24 aA
	MN	2.58 ± 0.15 bB	1.47 ± 0.11 aA	1.91 ± 0.22 bB
	HN	2.02 ± 0.24 cC	1.22 ± 0.12 bB	2.00 ± 0.23 bB
**Nongda80**	LN	2.72 ± 0.26 aA	1.62 ± 0.21 aA	2.23 ± 0.29 aA
	MN	2.33 ± 0.35 bB	1.38 ± 0.74 aA	1.97 ± 0.08 bB
	HN	1.94 ± 0.15 cB	1.16 ± 0.12 bB	2.09 ± 0.18 aA
**Tangyu10**	LN	2.56 ± 0.14 aA	1.43 ± 0.15 aA	2.74 ± 0.34 aA
	MN	2.35 ± 0.22 aB	1.35 ± 0.14 aA	2.12 ± 0.21 bB
	HN	2.23 ± 0.10 bB	1.31 ± 0.13 bB	1.84 ± 0.12 cC

Note: The statistical analyses are based on each cultivar. With respect to each cultivar, the values in the same column followed by different uppercase and lowercase letters are significantly different at the 0.01 and 0.05 probability levels, respectively. The corn cultivars were subjected to low (LN, 0 kg N ha^−1^), medium (MN, 75 kg N ha^−1^), and high (HN, 150 kg N ha^−1^) levels of N application over the cropping season (2002 and 2003). The data are average values across 2 years with ± one standard error.

**Table 5:** Correlation coefficients (r) among the measured parameters

parameters	LDW	F_v_/F_m_	Ph	LNC	LSC	S/N
LDW	1					
F_v_/F_m_	0.909**	1				
Ph	0.704**	0.577**	1			
LNC	0.698**	0.636**	0.671**	1		
LSC	0.432*	0.618**	0.704**	0.552**	1	
S/N	−0.546**	−0.798**	−0.714**	−0.886**	−0.408*	1

Note: LDW, leaf dry weight; F_v_/F_m_, the maximum quantum yield of PSII; Ph, photosynthetic CO_2_ assimilation; LNC, leaf nitrogen concentration; LSC, leaf sugar concentration; S/N, the sugar-to-nitrogen concentration ratio.

Probability levels are indicated by * and ** for 0.05, and 0.01, respectively.
